# Murine infection with bioluminescent *Leishmania infantum* axenic amastigotes applied to drug discovery

**DOI:** 10.1038/s41598-019-55474-3

**Published:** 2019-12-12

**Authors:** David Mendes Costa, Pedro Cecílio, Nuno Santarém, Anabela Cordeiro-da-Silva, Joana Tavares

**Affiliations:** 1 i3S – Instituto de Investigação e Inovação em Saúde, Universidade do Porto, Porto, Portugal; 2 IBMC – Instituto de Biologia Molecular e Celular, Universidade do Porto, Porto, Portugal; 3 Departamento de Ciências Biológicas, Faculdade de Farmácia da Universidade do Porto, Porto, Portugal

**Keywords:** Parasitic infection, Experimental models of disease

## Abstract

Leishmaniasis is an important vector-borne neglected tropical disease caused by *Leishmania* parasites. Current anti-*Leishmania* chemotherapy is unsatisfactory, justifying the continued search for alternative treatment options. Herein, we demonstrate that luciferase-expressing *Leishmania infantum* axenic amastigotes, unlike promastigotes, are highly infectious to BALB/c mice and thus generate a robust bioluminescent signal in target organs, such as the liver and the spleen, as early as two weeks after infection. Treatment with the reference drugs amphotericin B and miltefosine was effective at reducing parasite burdens. This model allows the assessment of treatment efficacy using whole-mouse bioluminescence imaging without the need to wait several weeks for spleen infections to be detectable by this non-invasive method. In conclusion, we propose the use of this model in an initial approach to evaluate the treatment efficacy of promising chemical entities without having to sacrifice large numbers of animals or to wait several days for a readout.

## Introduction

Leishmaniasis is a vector-borne parasitic disease caused by over 20 *Leishmania* species^1^. It affects approximately 12 million people worldwide, with up to 1 million new cases every year^1^. Visceral leishmaniasis (VL), the most severe form of the disease, is fatal if left untreated. VL is mainly associated with *Leishmania infantum* (New and Old Worlds) or *Leishmania donovani* (Old World) infections, as these parasites are capable of disseminating to the host internal organs, particularly the liver, spleen and bone marrow^1,2^. As there is still no vaccine available for humans, disease control relies mostly on chemotherapy and vector control. Therefore, the limited and unsatisfactory chemotherapeutic options dictate the need for new drugs^3,4^. Indeed, every year up to 30,000 individuals suffering from VL die, some of them due to clinical treatment failure (associated with drug resistance, drug quality/misuse and infection status)^1,5^. Fortunately, neglected tropical diseases such as leishmaniasis have become a relevant part of the global health agenda, with a consequent increase in investment in control strategies^6^. New leads against leishmaniasis are currently being optimized, while other compounds are already in pre-clinical and clinical stages^7,8^. Moreover, the recent development of *in vitro* high-throughput screening programs is expected to feed the anti-*Leishmania* drug discovery pipeline with new compounds^8–10^, whose efficacy will need to be addressed *in vivo*. Direct parasite observation remains the gold standard readout of anti-*Leishmania* drug efficacy *in vivo*^11^. However, the traditional parasitological methods used to this end (microscopic analysis of organ biopsies or limiting dilution assays) exhibit some limitations. Besides being labor-intensive and time-consuming, these methods only allow a static evaluation of infection since target organ collection entails euthanasia of the animal^8,11^. Indeed, the requirement of a large number of animals represents not only a technical but also an ethical limitation^8^. *In vivo* imaging techniques, namely those using bioluminescence-based models, have been developed to overcome such drawbacks^12^. Whole-body bioluminescence imaging has been aplied to detect *L. infantum* infection in mice^13–15^ and hamsters^16,17^. The latter develop similar clinicopathological features of VL in humans but several restrictions associated with their use, which include cost and the prolonged time of infection until symptoms appear, support the use of the mice model. In fact, mice are the most comonly used model to evalute the anti-leishmanial activity of promising chemical entities in an initial approach. Infections with *L. infantum* expressing firefly luciferase have been described and validated to study drug efficacy *in vivo*^13–15^. However, in these models, more than a month post-infection is normally required to generate robust bioluminescent signal in the spleen^13–15^. Mouse infections with *L. infantum* are commonly performed by injecting promastigotes, the flagellated extracellular parasite stage transmitted to the mammalian host following the bite of an infected sandfly vector. In the mammalian host, promastigotes are captured by phagocytic cells and transform into non-motile amastigotes inside the phagolysosome. The diferentiation from promastigotes to amastigotes is triggered by elevated temperature and drop in pH^18^. These conditions can be reproduced *in vitro* for culturing several *Leishmania* species^19,20^, including *L. infantum*, as axenic amastigotes^21^. These forms efficiently infect macrophages and have been used to identify compounds that are active against the intracellular *Leishmania* stage^22–24^. In this work, we investigated the infectivity of luciferase-expressing *L. infantum* axenic amastigotes in BALB/c mice. We found that this parasite form is highly infectious and generates a robust bioluminescent signal in target organs suitable for the evaluation of treatment efficacy using whole-mouse live imaging.

## Results and Discussion

**Figure 1:**
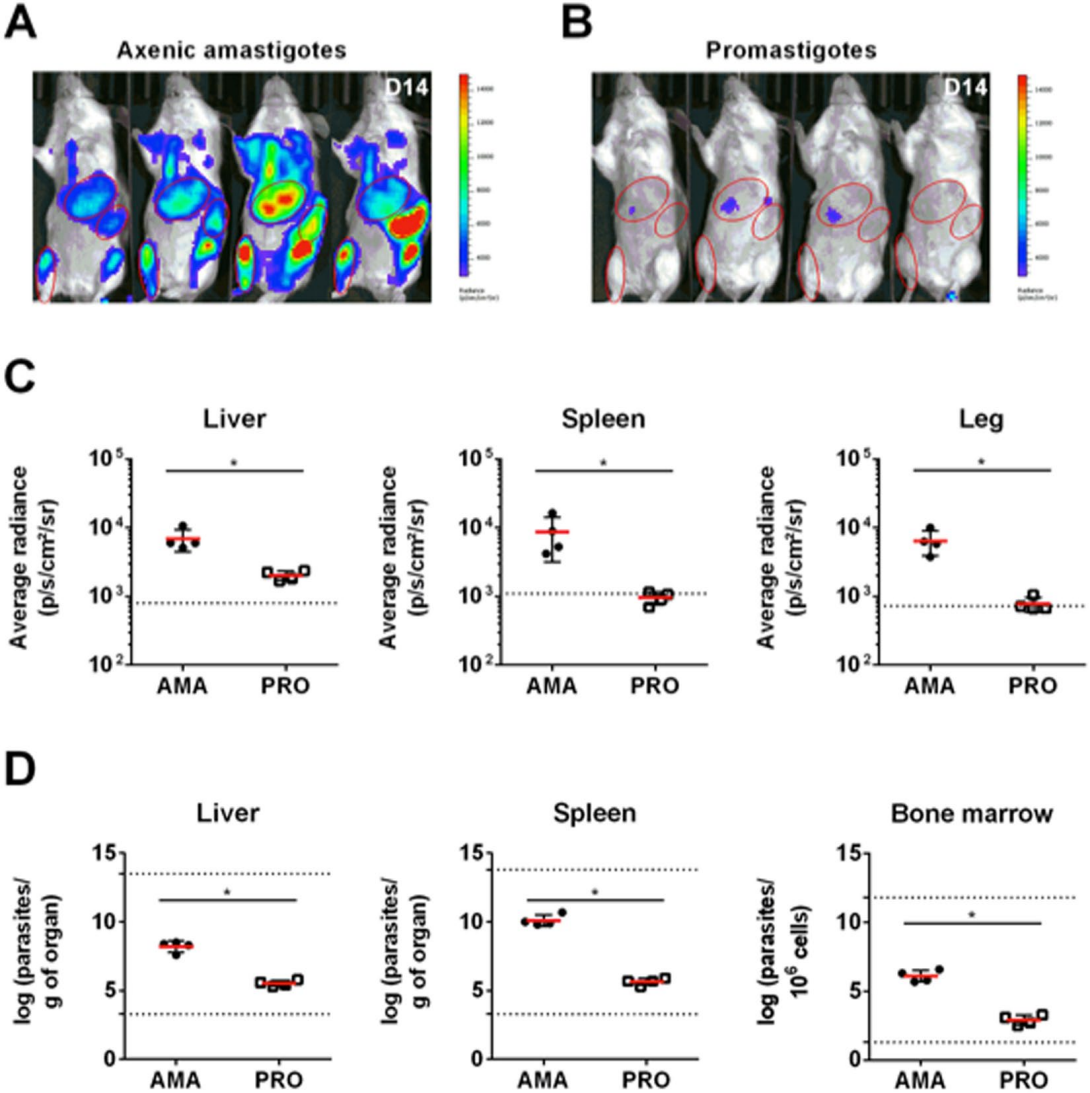
Infectivity of luciferase-expressing *L. infantum* axenic amastigotes and promastigotes via intravenous injection. (**A**,**B**) Images of BALB/c mice infected with either luciferase-expressing *L. infantum* axenic amastigotes (**A**) or promastigotes (**B**) resulting from the superimposition of the bioluminescence signal map and a grey-scale photograph of the mice. The regions of interest (ROIs) shown were used to quantify the bioluminescence signal originating from the liver, spleen, and right hind leg of the mouse. (**C**) Bioluminescence measurements expressed as average radiance (photons/s/cm^2^/steradian) corresponding to the previously defined liver (left), spleen (center), and right hind leg (right) ROIs. Means ± standard deviations are represented in bars. The dotted line represents the background signal calculated by applying the ROIs on images of uninfected animals. (**D**) Parasite burden in the liver, spleen, and femur bone marrow determined by limiting dilution 14 days post-infection. Means ± standard deviations are represented in bars. The dotted lines represent the upper and lower detection limit of the technique for each organ. (**C,D**) AMA: animals infected with 10^8^ axenic amastigotes. PRO: animals infected with 10^8^ promastigotes. Statistical significance calculated by Mann Whitney test: (*)*p* < 0.05. Data representative of two independent experiments.

In a previous study, we demonstrated that luciferase-expressing *L. infantum* axenic amastigotes^22^ injected intravenously generate a robust bioluminescent signal in mice on the first day of infection^25^. To assess if this signal could still be detected at later time points post-infection, thus allowing the assessment of treatment efficacy *in vivo*, we infected BALB/c mice with 10^8^ *L. infantum* axenic amastigotes or promastigotes intravenously (IV) (Fig. 1). Mice were then imaged 14 days post-infection (Fig. 1A) using an IVIS Lumina LT (PerkinElmer), 10 minutes after the administration of luciferin. The ventral fur was shaved to maximize photons detection and the mice placed in the dorsal position were angled to increase the detection of the signal coming from the spleen. The spatial distribution of the bioluminescent signal indicates parasite establishment in the anatomical regions encompassing target organs such as the liver, spleen, lymph nodes and bone marrow (Fig. 1A). Interestingly, mice infected with the same inoculum of *L. infantum* promastigotes exhibited almost no detectable bioluminescent signal (Fig. 1B). Using the Living Image software, elliptical regions (ROIs) were drawn to quantify bioluminescent signal in the anatomical regions of the liver, spleen, lymph nodes and bone marrow (the last two inferred from the signal of the left leg; Fig. 1A,B). The bioluminescent signal evaluated by the average radiance (photons/second/cm^2^/steradian) of the above ROIs was significantly higher in the animals infected with axenic amastigotes than in animals infected with promastigotes (Fig. 1C). Indeed, the signal in the spleen and leg of the animals infected with promastigotes was below the detection limit (Fig. 1C), which corresponds to the signal obtained when the above ROIs were applied to the images generated in the non-infected mice following luciferin administration.

**Figure 2:**
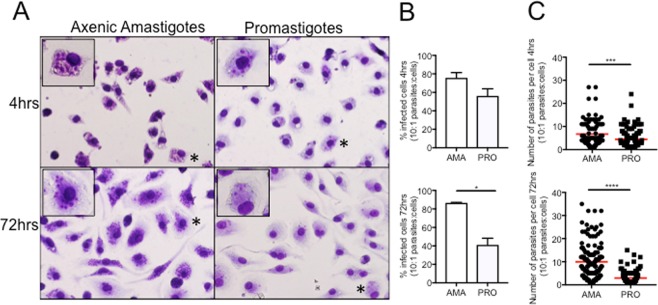
*In vitro* infectivity of *L. infantum* axenic amastigotes and promastigotes. (**A**) Images of peritoneal macrophages infected with either luciferase-expressing *L. infantum* axenic amastigotes (left) or promastigotes (right) at 4 hours or 72 hours post-infection and stained with Hemacolor. Asterisks indicate the magnified cell in the upper left corner of the image **(B)** Percentage of cells infected at 4 hours or 72 hours after infection. Data represented in bars corresponds to mean values + standard deviation represented in bars of experiment replicates. **(C)** Number of parasites per infected cell at 4 hours or 72 hours after infection. Each symbol represents the number of parasites per individual cell. Data from experiment replicates was pooled together. (**B, C**) AMA: infections were performed with axenic amastigotes. PRO: infections were performed with promastigotes. Statistical significance calculated by unpaired Student’s t test: (*)*p* < 0.05, (***)*p* < 0.001, (****)*p* < 0.0001. Data representative of two independent experiments.

To evaluate whether the differences in the bioluminescent signal detected in amastigote- and promastigote-infected mice were due to distinct infective capacities, parasite burdens in the liver, spleen and bone marrow were evaluated using the gold standard limiting dilution assay^26^. In fact, promastigote infection yielded significantly lower parasite burdens in these organs when compared to infections performed with axenic amastigotes (Fig. 1D). This indicates that the difference in the signal intensity was most likely due to a reduced number of parasites in these organs. Interestingly, *in vitro* data demonstrates that axenic amastigotes have an increased capacity to originate persistent infections in macrophages when compared with the promastigotes (Fig. 2).

This was observed using *in vitro* infections of peritoneal macrophages from BALB/c mice with axenic amastigotes and promastigotes at a ratio of 2 (data not shown) and 10 parasites per cell (Fig. 2). Both parasite forms were capable of infecting a similar percentage of macrophages as no significant differences were seen at 4 hours post-infection (Fig. 2A,B). However, the cells infected with axenic amastigotes were more parasitized when compared to promastigotes infections (Fig. 2A–C). This difference increases with time and at 72 hours post-infection the mean value of parasites per cell is ~10 versus ~3 for cells infected with axenic amastigotes or promastigotes respectively. Ultimately, this increased capacity of axenic amastigotes to originate infections that persist inside macrophages may contribute to their higher infectivity *in vivo*.

**Figure 3:**
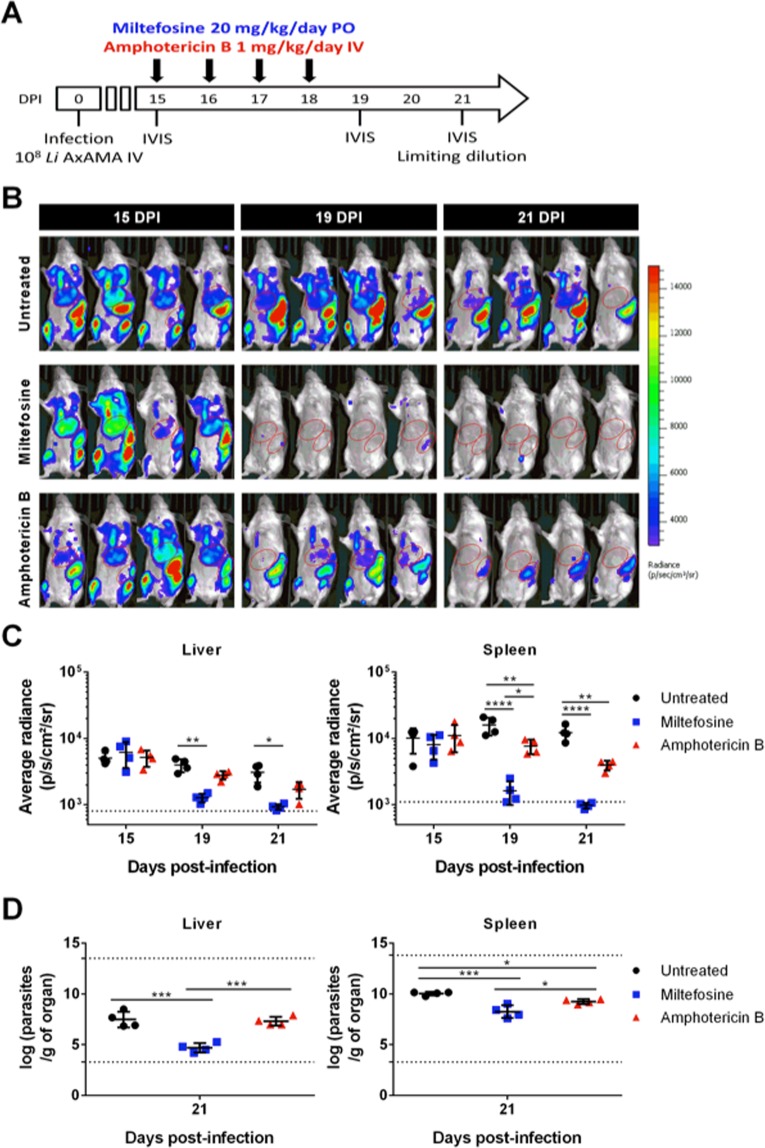
Treatment of *L. infantum* axenic amastigote-infected mice with reference drugs miltefosine and amphotericin B. (**A**) Schematic representation of the experimental design. BALB/c mice were infected with 10^8^ luciferase-expressing *L. infantum* axenic amastigotes (AxAMA) IV and 4-day treatments with either 20 mg/kg/day of miltefosine *per os* (PO) or 1 mg/kg/day of Amphotericin B (IV) were initiated 15 days post-infection (DPI). All animals (n = 4 per group) were imaged right before (day 15 post-infection), one day after (day 19 post-infection) and 3 days (day 21 post-infection) after the end of treatment using the IVIS Lumina LT system. At the last time point animals were sacrificed and parasite burden in the liver, spleen, and femur bone marrow were determined by limiting dilution. (**B**) Images of infected mice resulting from the superimposition of the bioluminescence signal map and a grey-scale photograph of the mice. The ROIs shown were used to quantify the bioluminescence signal originating from the liver and spleen anatomical regions. (**C**) Bioluminescence measurements expressed as average radiance (photons/s/cm^2^/steradian) corresponding to the previously defined liver (graph on the left) and spleen (graph on the right) ROIs. Means ± standard deviations are represented in bars. The dotted line represents the background signal calculated by applying the ROIs on images of uninfected animals. Statistical significance calculated by two-way ANOVA: (*)*p* < 0.05, (**)*p* < 0.01, (****)*p* < 0.0001. (**D**) Parasite burden in the liver (graph on the left) and spleen (graph on the right) determined by limiting dilution 21 days post-infection. The dotted lines represent the upper and lower detection limit of the technique for each organ. Means ± standard deviations are represented in bars. Statistical significance calculated by ordinary one-way ANOVA: (*)*p* < 0.05, (***)*p* < 0.001. Data representative of two independent experiments.

**Figure 4:**
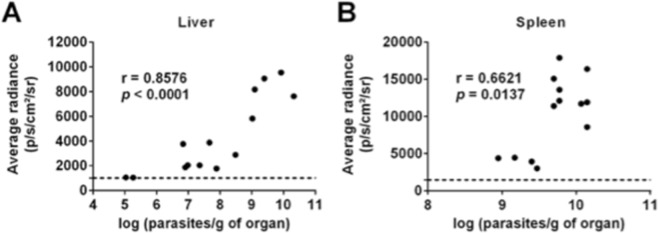
Relation between bioluminescence signal and parasite burden measured by limiting dilution. Average radiance (photons/s/cm^2^/steradian) from liver (**A**) or spleen (**B**) ROIs plotted against the matching parasite burden in the corresponding organ. Pooled data of individual mice and from two independent experiments is shown. The dashed line represents the upper limit of the 99% confidence interval of the mean average radiance values obtained for each ROI when applied on images of uninfected BALB/c mice (n = 6). Only animals displaying average radiance levels above the dashed line were considered for the calculation of the Pearson’s correlation coefficients using GraphPad Prism 6.0 version.

Considering that animals infected with axenic amastigotes yielded an early and sustained detectable bioluminescent signal in the main target organs, we tested the potential of this model to evaluate, using whole-mouse imaging, the effectiveness of *in vivo* treatments against *L. infantum*. Infected animals were treated with miltefosine at 20 mg/kg/day (*per os*) or amphotericin B at 1 mg/kg/day (IV) for four days starting from day 15 post-infection (Fig. 3A). This time-point was chosen due to a compromise between bioluminescence signal and parasite establishment in the target organs (Supplementary Figure 1A,B; splenic burdens plateau from 15 days post-infection, while signal in the liver is reproducibly detected at this time-point). Imaging was performed right before treatment (day 15 post-infection), and one (day 19 post-infection) and three days (day 21 post-infection) after the last day of treatment (Fig. 3B). On day 21 post-infection mice were sacrificed and the liver, spleen and bone marrow were harvested for parasite burden assessment by limiting dilution. As anticipated, the short miltefosine treatment was sufficient to significantly decrease the bioluminescent signal in the ROIs defined for the liver and spleen (Fig. 3C). Amphotericin B was not as effective, although a statistically significant difference was still obtained in the spleen when compared to the untreated animals. Similar results were observed when the parasite burden was determined by the limiting dilution method (Fig. 3D). However, parasites were still detected in animals whose bioluminescent signal was below background levels (Fig. 3D). Therefore, despite lower sensitivity, whole-animal bioluminescence imaging enabled the determination of the effectiveness of different treatments in reducing spleen and liver parasite burdens. We further evaluated the correlation between the two techniques used to determine parasite burdens in the liver and the spleen (Fig. 4). Average radiance values superior to the 99% confidence interval (GraphPad Prism 6.0 version) of the mean of the signal emitted by uninfected animals were plotted against the respective number of parasites per gram of liver (Fig. 4A) or spleen (Fig. 4B). Statistical significance, which translates into a positive correlation (GraphPad Prism 6.0 version), was found for both the liver and spleen determinations, evidencing the validity of this *in vivo* model.

Interestingly, while splenic parasite burdens remain constant up to at least week 14, as evaluated by either bioluminescence imaging or limiting dilution assay (Supplementary Figure 1A,B), liver burdens decrease to bioluminescent background levels as observed at eight weeks post-infection, suggesting the host could be controlling the infection in this organ (Supplementary Figure 1A)^27^, as it is observed in other murine models of visceral disease due to the known organ-specific immunity^28^.

In conclusion, we demonstrate the superior infectivity of *L. infantum* axenic amastigotes compared to promastigotes. This superior infectivity has also been reported for other *Leishmania* species either responsible for cutaneous (*L. mexicana* and *L. major*)^29,30^ or visceral infection (*L. donovani*)^30,31^. We propose the use of this rapid bioluminescence-based model for a time and cost effective preliminary *in vivo* testing of promising compounds against *L. infantum*. This minimally invasive method not only allows the assessment of treatment efficacy but also enables the adjustment of treatment regimens in a simple approach without the need to sacrifice large numbers of animals or to wait several days for a reliable readout. We expect this model to be a useful addition to the tools available to assist in the search for novel drugs to treat VL.

## Materials and Methods

### Ethics statement

The Instituto de Biologia Molecular e Celular.Instituto Nacional de Engenharia Biomédica Animal Ethics Committees approved all the experiments carried out on mice and the project was licenced by the Portuguese National Authority for Animal Health, in accordance to the statements on the directive 2010/63/EU of the European Parliament and Council.

### Mice and parasites

Six- to seven-week-old BALB/c mice were purchased from Charles River or the i3S animal facility. The *L. infantum* line used in this study is a clonal line (MHOM/MA/67/ITMAP-263) with an episomal expression of firefly luciferase under the control of the alpha-tubulin intergenic region (pGMαNEOαLUC vector)^22^. Both promastigotes and amastigotes were cultured under drug pressure (60 μg/ml of neomycin) in complete RPMI 1640 at 27 °C or complete cell-free medium MAA at 37 °C with 5% CO2, respectively^25,32^. Axenic amastigotes were differentiated from promastigotes, which in turn were derived from spleen cells recovered from infected BALB/c mice, to maintain parasite infectivity^32^. Briefly, a single high-density subpassage was performed and after five days these promastigotes were transformed into axenic amastigotes^22^. For the infections performed in this work, axenic amastigotes and promastigotes were cultured for a maximum of six subpassages.

### Mice infections, treatments and parasite load quantification

Promastigotes or axenic amastigotes recovered from a five days old cultures were washed twice with PBS and injected IV in mice (10^8^ parasites in 100 ul PBS). Treatments were performed starting from day 15 post-infection for four consecutive days with miltefosine at 20 mg/kg/day (*per os*) or amphotericin B at 1 mg/kg/day (IV).

Parasite loads were evaluated using whole-mouse bioluminescent *in vivo* imaging (IVIS LUMINA LT, Perkin Elmer). Mice had their ventral fur shaved with an appropriate clipper and were anesthetised with 2.5% isoflurane prior to the subcutaneous injection in the neck of 2.4 mg of D-luciferin. After a ten minute incubation time to allow the distribution of the substrate in the body of the anesthetised animals, a ten-minute signal acquisition controlled by the Living Image software (Perkin Elmer) was initiated. At the end of this period, animals returned to their cage and recovered from the anaesthesia^33^.

Parasite loads were also determined by limiting dilution^26,33,34^. Briefly, organ homogenates (1 mg of spleen; 4 mg of liver) were subjected to two-fold serial dilutions in quadruplicate with Schneider’s Insect medium in 96-well microtitration plates. The plates were incubated at 27 °C for 15 days and then each well was inspected for the presence or absence of promastigotes. The number of parasites per gram of organ was calculated as previously^26,33,34^.

### Macrophage infections and parasite quantifications

Peritoneal macrophages were collected from BALB/c mice using ice-cold PBS. 2 × 10^5^ peritoneal cells were seeded in 8-well chamber slides (LabteK) and cultured in complete RPMI at 37 °C, 5% CO2. The day after, unattached cells were removed and infections were performed with 2 × 10^6^ promastigotes or axenic amastigotes (corresponding to a 10:1 ratio parasites:cells) in complete RPMI for 4 hours at 37 °C, 5% CO2. Cells were then washed three times with pre-warmed medium to remove extracellular parasites. Cells were either fixed (for the 4 hours post-infection time point) or cultured for 3 additional days. Methanol fixed cells were stained with Hemacolor. The slides were analysed with an Olympus BX63 microscope and the images were taken using the Olympus cellSens software. The percentage of infected cells were determined by counting at least 300 cells per replicate. The number of parasites per infected cell was calculated by counting at least 50 infected cells per replicate.

### Statistical analysis

All statistical analyses and graphical representations of the data were performed using GraphPad Prism 6 software. Mann Whitney, one- or two-way analysis of variance (ANOVA) and Student’s t-test were performed and statistical significance was asserted whenever the p value was lower than 0.05 and represented by *(p < 0.05),**(p < 0.01),***(p < 0.001) and ****(p < 0.0001).

## Supplementary information

Supplementary_1

## Data Availability

All data generated or analysed during this study are included in this published article and its Supplementary Information files.
