# Supplementary material for: Murine infection with bioluminescent Leishmania infantum axenic amastigotes applied to drug discovery
Source: Sci Rep. 2019 Dec 12;9:18989. doi: 10.1038/s41598-019-55474-3 (PMC6908656; doi:10.1038/s41598-019-55474-3)
Supplement: Supplementary file 1 — Supplementary_1 [file 41598_2019_55474_MOESM1_ESM.pdf]

# Murine infection with bioluminescent *Leishmania infantum* axenic amastigotes applied to drug discovery

David M. Costa[1,2], Pedro Cecílio [1,2], Nuno Santarém[1,2], Anabela Cordeiro-da-Silva [1,2,3]* and Joana Tavares[1,2]*

[1] i3S – Instituto de Investigação e Inovação em Saúde, Universidade do Porto, Porto, Portugal;
[2] IBMC – Instituto de Biologia Molecular e Celular, Universidade do Porto, Porto, Portugal;
[3] Departamento de Ciências Biológicas, Faculdade de Farmácia da Universidade do Porto, Porto, Portugal;

*Corresponding authors: Joana Tavares (jtavares@ibmc.up.pt) and Anabela Cordeiro-da-Silva (cordeiro@ibmc.up.pt);

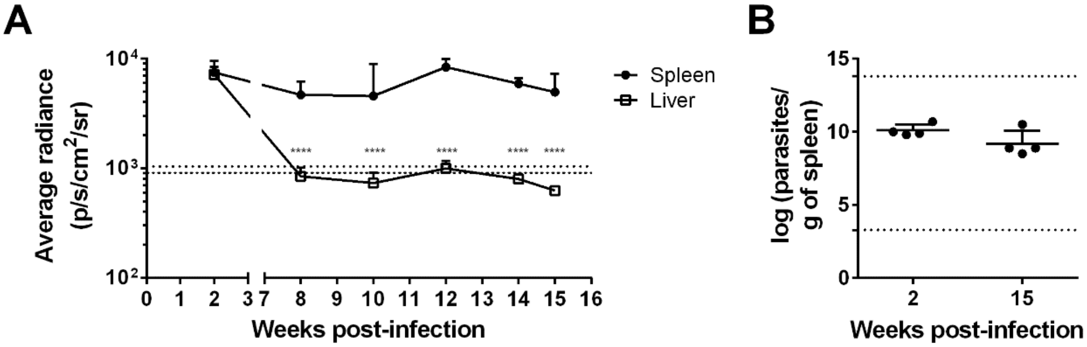


**Supplementary Figure 1**. Progression of parasite burdens in target organs following infection with *L. infantum* axenic amastigotes. (A) Bioluminescent signal in the ROIs corresponding to the spleen and liver anatomical regions of BALB/c mice intravenously infected with *L. infantum* axenic amastigotes in the first 15 weeks post-infection, expressed in average radiance (photons/s/cm$^2$/sr). Dotted and dashed lines represent the background average radiance levels for the spleen and liver ROIs, respectively. Means + standard deviations ($n = 4$) are represented in bars. Statistical significance versus the 2 weeks post-infection time point was calculated by two-way ANOVA: $p < 0.0001$ (****). (B) Parasite burdens in the spleen determined by limiting dilution 2 and 15 weeks post-infection. The dotted lines represent the upper and lower detection limit of the technique. Means + standard deviations are represented in bars. Statistical significance was assessed using the Mann Whitney test.
